# Diabetic Corneal Neuropathy

**DOI:** 10.3390/jcm9123956

**Published:** 2020-12-06

**Authors:** Hassan Mansoor, Hong Chang Tan, Molly Tzu-Yu Lin, Jodhbir S. Mehta, Yu-Chi Liu

**Affiliations:** 1 Al Shifa Trust Eye Hospital, Rawalpindi 44000, Pakistan; hassan-mansoor@hotmail.com; 2 Department of Endocrinology, Singapore General Hospital, Singapore 169608, Singapore; tan.hong.chang@singhealth.com.sg; 3 Tissue Engineering and Cell Therapy Group, Singapore Eye Research Institute, Singapore 169856, Singapore; molly.lin.t.y@seri.com.sg (M.T.-Y.L.); jodhbir.s.mehta@singhealth.com.sg (J.S.M.); 4 Cornea and External Eye Diseases, Singapore National Eye Centre, Singapore 168751, Singapore; 5 Eye-Academic Clinical Program, Duke-National University Singapore Graduate Medical School, Singapore 169857, Singapore

**Keywords:** corneal nerves, diabetic keratopathy, diabetes mellitus, diabetic neuropathy, neurotrophic keratopathy

## Abstract

Diabetic keratopathy (DK) is a common, but underdiagnosed, ocular complication of diabetes mellitus (DM) that has a significant economic burden. It is characterised by progressive damage of corneal nerves, due to DM-induced chronic hyperglycaemia and its associated metabolic changes. With advances in corneal nerve imaging and quantitative analytic tools, studies have shown that the severity of diabetic corneal neuropathy correlates with the status of diabetic peripheral neuropathy. The corneal nerve plexus is, therefore, considered as an important surrogate marker of diabetic peripheral neuropathy and helps in the evaluation of interventional efficacy in the management of DM. The clinical manifestations of DK depend on the disease severity and vary from decreased corneal sensitivity to sight-threatening corneal infections and neurotrophic ulcers. The severity of diabetic corneal neuropathy and resultant DK determines its management plan, and a step-wise approach is generally suggested. Future work would focus on the exploration of biomarkers for diabetic corneal neuropathy, the development of new treatment for corneal nerve protection, and the improvement in the clinical assessment, as well as current imaging technique and analysis, to help clinicians detect diabetic corneal neuropathy earlier and monitor the sub-clinical progression more reliably.

## 1. Introduction

Diabetes mellitus (DM) is a chronic metabolic disorder, that is characterised by the increase in blood glucose, due to insulin deficiency from the destruction and loss of insulin-producing pancreatic b-cells (Type 1) or as a consequence of both insulin resistance and “relative” impairment in insulin secretion (Type 2) [1]. Currently, 415 million adults worldwide are diagnosed with DM, and more than 640 million people are projected to develop DM by 2040 [1]. Consequently, the economic burden of DM is estimated to increase in tandem, from $1.3 trillion in 2015 to $2.1 trillion by 2030 [2,3]. Such predictions constitute an enormous public health challenge and economic burden that many countries will face in the coming years, and further highlight the importance of preventing the various complications linked to DM.

DM is associated with macrovascular complications, such as cardiovascular disease, as well as microvascular complications, like retinopathy, nephropathy and neuropathy [4,5]. Diabetic retinopathy is one of the most frequently cited and well-studied complication associated with DM, but uncontrolled DM can affect other parts of the eye, and this is often overlooked. As a result, eye care professionals often miss the impact of DM on the anterior segment of the eye like cornea, conjunctiva, lacrimal glands and lens that can also lead to severe visual loss and even blindness [6]. Diabetic keratopathy (DK) affects 47–64% of patients during the clinical course of DM, and it is mostly underdiagnosed [7]. In DK, there is progressive damage of corneal nerves that decreases the corneal sensitivity and increases the risk of anterior segment pathological conditions ranging from dry eye disease to neurotrophic ulcers with a risk of sight-threatening corneal infections [8]. In the Italian National Health Service, the mean annual treatment cost of neurotrophic keratopathy (NK) per patient can vary from €5167 (persistent epithelial defect) to €10,885 (corneal ulcer without perforation) [9]. The diverse clinical presentations and significant economic burden of DK encourage clinicians and researches to explore neuronal changes in diabetic corneas further.

In this review, we will discuss the impact of DM on corneal nerve plexus, its clinical implications and assessment, and the association between diabetic corneal neuropathy and diabetic peripheral neuropathy (DPN). We will probe the neurotrophic role of corneal nerve fibres in maintaining corneal microenvironment and promoting wound healing, as well as exploring available biomarkers, along with neuroprotective therapies for diabetic corneal neuropathy.

## 2. Pathogenesis of Diabetic Neuropathy and Diabetic Corneal Neuropathy

When DM is not well treated, chronic hyperglycaemia ensues, and this leads to a variety of metabolic changes, such as the accumulation of advanced glycation end products (AGE), increased polyol pathway flux, reactive oxygen species (ROS) production, as well as activation of protein kinase C (PKC) pathway. These metabolic changes complement each other through a complex interplay to cause diabetic neuronal degeneration, which results in diabetic neuropathy [1]. Like other peripheral nerves, DM could also adversely affect corneal nerves [10]. The mechanism of impaired corneal innervation in diabetic individuals is regarded to be the same as the pathogenesis of diabetic neuropathy described below. In support of this, overexpression of AGE, 8-hydroxydeoxyguanosine (a marker of oxidative stress) and nuclear factor-kappa B (NF-κB; (AGE-mediated downstream signal pathway involved in ROS production and the release of pro-apoptotic cytokines) were found in diabetic corneas in rats, suggesting that these factors may be implicated in the development of diabetic corneal neuropathy by causing corneal neuronal degeneration and apoptosis of neural cells [11].

### 2.1. Accumulation of Advanced Glycation End Products (AGE)

Glycation has been described as the formation of a non-enzymatic covalent bond between a lipoprotein or protein and a sugar molecule, e.g., glucose or fructose, which leads to the creation of glycated proteins [12]. These glycated proteins undergo cross-linking over time to form functionally deranged modified protein products that are known as advanced glycation end products (AGE) [1]. In DM, glycation affects the myelin proteins of peripheral nerves [13]. Accumulation of AGE has been observed in the perineural collagen, Schwann cells and axoplasm of diabetic nerves, causing axonal degeneration and functional alteration [14].

### 2.2. Polyol Pathway

The polyol pathway, also known as the sorbitol-aldose reductase pathway, is well known for its role in the pathogenesis of diabetic neuropathy. During hyperglycaemia, excess glucose in the nerve cells is shunted to the polyol pathway, as the normal glycolytic pathway becomes saturated [15]. Subsequently, intracellular enzymes, e.g., aldose reductase and sorbitol dehydrogenase convert the excess glucose to sorbitol and fructose, which become accumulated within the nerve cells because the nerve cell membrane is impermeable to both sugar molecules [16]. Consequently, the osmotic stress in the nerve cell increases, whereas the level of free nerve myoinositol decreases [1]. The myoinositol is a sugar alcohol that is important for normal nerve conduction velocity [17]. A reduction in its level results in a decreased membrane sodium-potassium adenosine triphosphatase (Na+/K+ ATPase) activity, thereby leading to electrolyte imbalance together with abnormal function and structural breakdown of nerve fibres [1].

### 2.3. Oxidative Stress

Oxidative stress refers to a cellular environment in which the ROS production is elevated, and the scavenging capacity of antioxidants are unable to reduce ROS to non-toxic substances [18]. During hyperglycaemia, the oxidative metabolism of surplus sugar molecules in the mitochondria increases, leading to the generation of superoxide ions, a sub-family of ROS. The elevated production of superoxide ions coupled with decreased reducing capacity of antioxidant enzymes causes a critical imbalance in the mitochondrial electron transport chain [19]. Nerve fibres have a sizeable mitochondrial volume, and therefore, are more prone to hyperglycaemia-induced mitochondrial damage. The resultant mitochondrial injury may decrease the available cellular energy, and as a result, nerve conduction blockage and axonal demyelination ensue [1,20]. Although oxidative stress plays a vital role in the pathogenesis of diabetic neuropathy, effective antioxidant therapies to prevent or delay the development of diabetic neuropathy remain an area of ongoing research.

### 2.4. Protein Kinase C (PKC) Activation Pathway

Researchers and clinicians have explored the role of PKC as a causative mechanism of diabetic neuropathy. In diabetic patients, intracellular hyperglycaemia promotes the synthesis of diacylglycerol, which is an activator of PKC. The activation of PKC affects Na+/K+ ATPase activity that impairs neuronal conduction and regeneration [21]. Recently, PKC inhibitors have gained increased attention, as they have the potential to improve nerve conduction and alleviate symptoms of diabetic neuropathy [22]. In a randomised-controlled trial, isoform-selective PKC-beta inhibitor ruboxistaurin mesylate improved the endothelium-dependent and C fibre-mediated microvascular blood flow in the skin, as well as the nerve conduction, neurological deficits in the upper and lower extremities, and the quality of life in type 1 and type 2 diabetic patients [22].

## 3. Diabetic Peripheral Neuropathy (DPN)

Diabetic peripheral neuropathy (DPN) is a common systemic complication of DM that affects about 50% of diabetic patients [23]. It is characterised by a progressive loss of small and large peripheral nerve fibres in a distal to proximal fashion, which may lead to decreased sensitivity or complete absence of sensations in the affected region [24]. As researchers and clinicians are striving to identify potential neuroprotective and neuro-regenerative therapies to prevent or even reverse established DPN, efforts are also underway for early detection of DPN. By doing so, DPN-related sequelae, such as lower extremity ulceration or gangrene, which could lead to amputation of the affected region, is prevented [25].

Currently, there is no sensitive, non-invasive and reproducible diagnostic method to measure small peripheral nerve fibre changes in diabetic patients. Several diagnostic modalities, such as clinical assessment of neurological signs and symptoms, quantitative sensory testing (QST), and nerve conduction studies (NCS) have been used for the evaluation of DPN, but vibration perception assessment using biothesiometry is regarded as the gold standard for it [1,26]. Limitations associated with the aforementioned diagnostic tools include (1) Variability in diagnosis and overestimation of DPN [27] (2) Inability to perform accurate and reproducible QST in non-cooperative or non-attentive subjects [28] (3) Inability of NCS and biothesiometry to measure small nerve fibre function, which is an earlier indicator of DPN [29,30]. Although skin punch biopsy has been proposed as the objective evaluation of the morphology of small nerve fibres in DPN, it lacks a functional assessment and is not suitable for repeat testing [31]. Moreover, a skin punch biopsy is invasive and poses a risk of abnormal wound healing and infection [1].

In diabetic patients, the morphological impact of DM on corneal subbasal nerve plexus has been shown to correlate with peripheral nerve fibre changes [1,10]. Further, symptoms and the severity of DPN are linked with progressive loss of corneal nerve fibre density (Section 4.2) [1,32,33]. Hence, evaluation of corneal innervation in diabetic patients has garnered considerable attention and has been proposed as a surrogate marker for small nerve fibre neuropathy in DM [34,35].

## 4. Diabetic Corneal Neuropathy

### 4.1. Corneal Innervation

The human adult cornea comprises of five layers and is about 550 μm in thickness. While the outermost layer is the corneal epithelium that is followed by Bowman’s membrane, corneal stroma, Descemet’s membrane, the innermost layer consists of corneal endothelial cells. The cornea is the most densely innervated tissue in the human body. It has a central nerve density of about 7000 nociceptors/mm^2^ and is reported to be 300–600 times more sensitive than the skin [36]. Although the sensory innervation of the cornea is provided by a nasociliary branch of the ophthalmic division of the trigeminal nerve via 70–80 long ciliary nerves, the superior cervical ganglion supplies sympathetic innervation to it [37].

Anatomically, 70 to 80 large diameter myelinated nerves enter the peripheral cornea at the corneoscleral limbus at the level of posterior to mid-stroma. Subsequently, they run radially and anteriorly towards the central cornea, where they give rise through repetitive branching to multiple progressively smaller unmyelinated nerve fibres. These unmyelinated nerve fibres are thick and eventually form a mid-stromal nerve plexus, which innervates the anterior stromal layers via its distal branches. The corneal nerves become progressively thinner as they extend to the anterior-most layers of the cornea. The width of the corneal nerves in the anterior stroma (5.2 ± 1.7 μm) is less than the width of the nerves in the mid-stroma (6.3 ± 1.8 μm) [38].

**Figure 1:**
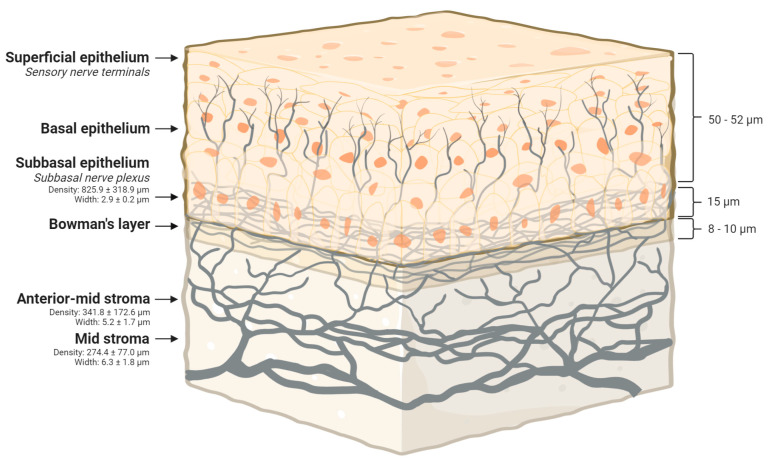
A three-dimensional section of the human cornea depicting the corneal nerve density and width at different layers. The figure has been created with BioRender.com.

The distal branches of mid-stromal nerve plexus later continue into the anterior stroma located immediately beneath the Bowman’s membrane and give rise to a subepithelial plexus that is denser than the mid-stromal nerve plexus (density; 314.6 ± 153.5 μm and 274.4 ± 77.0 μm, respectively). While some nerve fibres of the subepithelial plexus terminate in the subepithelial stroma as free nerve endings, others pass through the Bowman’s membrane and give rise to very thin subbasal nerves (2.9 ± 0.2 μm) that anastomose extensively with one another to form a subbasal nerve plexus. The subbasal nerve plexus is the densest plexiform arrangement in the cornea (825.9 ± 318.9 μm) and lies between the Bowman’s layer and the basal cell layer of the corneal epithelium. In addition, the subbasal nerve plexus gives origin to intraepithelial nerve terminals of varied length, directional orientation, as well as anatomical complexities, which are distributed throughout the corneal epithelium (Figure 1) [39]. These intraepithelial nerve terminals have three main groups of receptors, e.g., chemical or polymodal nociceptors, thermal or cold receptors together with mechanical or mechanonociceptors that produce a sensation of pain from chemical, thermal and mechanical stimulation, respectively [38].

The morphology and distribution of corneal nerves can be visualised with different types of staining techniques. Conventional staining techniques, such as gold chloride, silver staining and methylene blue, are now less commonly used because they require a high level of technical skill. Also, their sensitivity to show nerve patterns is low [40]. Immunohistochemical staining with anti-class β III tubulin marker and acetylcholinesterase (AChE) staining are the two most commonly used techniques. The former is a marker for microtubule elements, which are found almost exclusively in neurons, and the latter exists in corneal nerve axons to maintain the ionic gradient during propagation of the nerve impulse along the axons [41]. Nonetheless, these staining techniques can only be used for ex-vivo corneas. Therefore, in-vivo confocal microscopy (IVCM), a non-invasive scanning modality providing detailed information on the corneal nerve plexus, has become a valuable tool and gold standard for ophthalmologists to evaluate the status of corneal nerve fibres (Section 5.2.5).

### 4.2. Corneal Nerve Plexus: A Surrogate Marker of DPN and Interventional Efficacy

The corneal subbasal nerve plexus lies in a single plane parallel to the corneal surface, and its density is, therefore, easier to be evaluated with IVCM compared to that of stromal nerves that are distributed throughout the stroma in a three-dimensional pattern [36]. Moreover, the corneal subbasal nerve plexus is the densest and most recognisable component of the corneal innervation; hence, it has been considered as an anatomical landmark to assess corneal nerve morphology. The high reproducibility of IVCM to evaluate the corneal subbasal nerve plexus quantitatively [42] makes it increasingly important for clinicians to assess corneal nerve changes objectively (Section 5.2.5).

The relationship between corneal neuropathy and DPN has been described. Petropoulos et al. reported that corneal nerve fibre density, branch density, and nerve fibre length differed significantly between control subjects and type 1 and type 2 diabetic patients, and the corneal nerve metrics worsened with increasing severity of DPN [43]. Furthermore, corneal nerve branch density and fibre length were reduced in diabetic patients with mild DPN in comparison to both controls and diabetic patients without DPN, suggesting that corneal nerve parameters could be used as markers for DPN assessment [35]. Similarly, the LANDMark study and a study conducted by Hertz et al. [32] concluded that the corneal nerve fibre length was predictive of DPN development and was a reproducible biomarker of early DPN [44]. The corneal nerve fibre damage in type 1 and type 2 diabetic patients was also significantly associated with decreased corneal sensitivity and pathological vibration sensation of the great toe [33]. Of note, the reduction in subbasal nerve plexus density and corneal sensitivity has been shown to precede other clinical and electrophysiology tests of DPN, supporting the important role of subbasal nerve plexus as a surrogate marker for DPN [45]. A meta-analysis that included 13 studies and 1680 participants supported the findings mentioned above and concluded that corneal nerve parameters reduced significantly in patients with DM, especially when the DM status was complicated with DPN, implying that corneal nerve morphology assessment is useful for the detection and evaluation of early nerve damage in diabetic patients [46].

The corneal nerve plexus has also been used as a biomarker to determine interventional efficacy in the management of DM. Corneal nerves have been shown to regenerate in diabetic individuals after adopting measures to improve glycaemic control, especially HbA1c [47,48]. In type 1 DM patients receiving pancreas-kidney transplantation, the corneal nerve fibre density, branch density and nerve fibre length improved at six months postoperatively, suggesting the role of corneal nerve plexus as a secondary outcome measure following this surgical intervention [47].

### 4.3. Neurotrophic Role of Corneal Nerves

Corneal nerves provide trophic support to various cells that constitute the cornea by secreting biologically active mediators, collectively known as neuromediators, including neurotrophins, neuropeptides, and neurotransmitters [49]. These neuromediators complement each other through a neurobiological interplay to maintain neuronal health, as well as promote neuronal regeneration and wound healing in DM. While sensory innervation of the cornea secretes neurotrophins and neuropeptides, autonomic innervation produces catecholamines and acetylcholine [1,36].

The neurotrophic role of corneal nerves maintains a healthy cornea and promotes corneal wound healing [37]. Several neurotrophic factors, such as neurotrophin-3 (NT-3) and nerve growth factor (NGF), are essential for maintaining neuronal health and promoting neuronal repair mechanisms [1,50]. NGF also promotes corneal wound healing by upregulating anti-inflammatory pathways that induce production of cytokines, e.g., interleukin (IL)-1 receptor antagonists and IL-10, which reduce neuroinflammation. The inflammatory stimulus causes increased expression of the NGF receptor Tropomyosin Receptor Kinase A (TrkA), and the subsequent binding of NGF to TrkA decreases NF-κB nuclear translocation, inhibits glycogen synthase kinase 3 (GSK3) activity, as well as enhances the activation of the phosphatidylinositol 3-kinase (PI3K)/Akt pathway that results in decreased production of inflammatory cytokines [51]. Similarly, ciliary neurotrophic factor (CNTF) provides neuroprotective function and promotes corneal epithelial wound healing by upregulating the activation of corneal epithelial progenitor cells [52]. The mRNA and protein levels of CNTF were significantly down-regulated in diabetic mice compared to those in normal mice, and supplementation of exogenous CNTF promoted corneal epithelial wound healing. In streptozotocin-induced type 1 diabetic mice, DM had a significant impact on the population and infiltration of dendritic cells (DCs), the major source of CNTF in the cornea, by disrupting their neural communications. Low levels of DC-associated corneal nerve trophic factors could, therefore, result in diabetic corneal neuropathy [53]. A detailed listing of the neurotrophic factors produced by corneal nerves and their functions is shown in Table 1.

DM causes a decrease in the concentration of neuromediators [1]. Hence the beneficial effects of neurotrophic factors on neural homeostasis, neuronal regeneration and wound healing are lost in diabetic patients, and it results in worsening of the existing diabetic neuropathy [1]. Although the understanding of the neurotrophic role of corneal nerves has evolved in recent years, further research is required to explore and validate the use of corneal neuromediators as adjuncts to aid neuronal regeneration and wound healing in patients with diabetic corneal neuropathy.

## 5. Clinical Manifestations and Evaluation of Diabetic Corneal Neuropathy

The clinical manifestations of diabetic corneal neuropathy vary with disease severity. NK is a manifestation of diabetic corneal neuropathy that may result in corneal hypoesthesia, dry eye, recurrent corneal erosions, non-healing corneal epithelial defects, neurotrophic ulceration and vision-threatening corneal infections [8].

### 5.1. Neurotrophic Keratopathy (NK)

**Figure 2:**
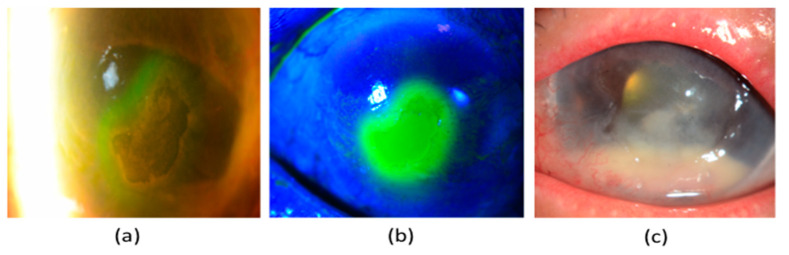
Clinical manifestations of neurotrophic keratopathy (NK). (**a**) A persistent oval corneal epithelial defect with smooth and rolled edges. (**b**) The neurotrophic ulcer has been stained with 2% *w*/*v* solution of fluorescein sodium (**c**) Secondary microbial infection of the neurotrophic corneal ulcer with a hypopyon in the anterior chamber.

NK is a disease that is related to alterations in corneal nerves, leading to impairment of their sensory and trophic functions, with a consequent breakdown of the corneal epithelium, as well as affecting health and integrity of the tear film, epithelium and stroma [54]. Its reported incidence in diabetic patients is 18% [62]. NK is characterised by three stages. Stage 1 NK is described by the presence of irregular corneal epithelium, superficial punctate keratopathy, dellen formation, and decreased tear break-up time. Moreover, persistent stage 1 NK may lead to corneal epithelial hyperplasia, stromal scarring, and superficial neovascularisation. In some patients, inferior bulbar conjunctival damage may also be observed by Rose-Bengal staining as the earliest sign. Stage 2 NK is characterised by a recurrent corneal epithelial breakdown or non-healing corneal epithelial defect with an oval or circular configuration (Figure 2a,b). The persistent corneal epithelial defect is often surrounded by a loose and oedematous corneal epithelium, which has a tendency to detach spontaneously. With time, the edges of the corneal epithelial defect become smooth and rolled. A corneal ulcer with stromal involvement that may be further complicated by stromal melting and corneal perforation usually manifests stage 3 NK. Additionally, an anterior chamber inflammatory reaction together with a hypopyon might be seen (Figure 2c) [63]. Of note, there may be a delayed diagnosis or under-diagnosis as these patients are often asymptomatic, due to decreased corneal sensation.

### 5.2. Evaluation of Diabetic Corneal Neuropathy

The clinical evaluation of a patient with diabetic corneal neuropathy involves taking a detailed history, corneal sensitivity assessment, slit-lamp biomicroscopy evaluation for the ocular surface integrity with vital stains and tear film function, as well as IVCM imaging on corneal nerves.

#### 5.2.1. Clinical History

The patient’s medical and surgical history should be probed to rule out conditions other than DM that could be associated with the development of NK. Other concurrent ocular manifestations specific to the aetiology would also help in the differential diagnosis. However, the essential components of clinical manifestations of diabetic corneal neuropathy are not different from those, due to other causes of NK that have been summarised in Table 2.

#### 5.2.2. Corneal Sensitivity Assessment

It is well known that corneal sensitivity decreases in diabetic patients and its degree of loss correlates with the severity of DM [8]. The evaluation of corneal sensitivity is important to aid the diagnosis of diabetic corneal neuropathy and to determine the severity of corneal neuronal impairment in these patients. A cotton wisp or thread could be used to measure the corneal sensitivity qualitatively. Normal subjects elicit a blink reflex and describe the touch sensation whenever the cotton wisp or thread touches the cornea. In patients with diabetic corneal neuropathy, either there is no reaction, or there is a diminished blink response depending upon the severity of corneal nerve impairment.

Corneal esthesiometers have been used widely in clinics for the quantitative assessment of corneal sensitivity [66]. Among them, Cochet-Bonnet esthesiometer is a commonly used device and consists of a nylon monofilament with a constant diameter. The length of the monofilament can be extended from 0 to 6 cm, and by doing so; the pressure exerted on the corneal surface by the monofilament is altered. While subjects show a blink response when the monofilament touches the cornea, the length of monofilament that stimulates a sensation or evokes a blink response represents the corneal touch threshold of the subjects and is a measure of their corneal sensitivity [26,63]. The Belmonte non-contact esthesiometer also provides a measurement in an objective and non-invasive manner. Unlike the Cochet-Bonnet esthesiometer, it allows separate assessment of corneal mechanical, chemical and thermal sensitivity thresholds by eliciting corneal stimulation through air puffs at different pressures, temperatures and concentrations of carbon dioxide [67].

#### 5.2.3. Ocular Surface Staining

Several stains are used to determine the integrity of the ocular surface. The most commonly used vital stain that aids in the identification of corneal and conjunctival epithelial damage is 2% *w*/*v* solution of fluorescein sodium (Figure 2b). Lissamine Green and Rose Bengal stains also evaluate ocular surface integrity in patients with NK by staining dead and devitalised cells, as well as mucous filaments [63]. However, Rose Bengal stain causes much more discomfort and toxicity than the Lissamine green stain, and its usage has been phased out. The ocular surface staining improves the ability to estimate the disease severity, decide subsequent treatment options, and monitor disease progression or response to treatment [68].

#### 5.2.4. Tear Film Function

The tear film plays an important role in maintaining the integrity of the ocular surface. Tear film dysfunction is commonly found in patients with DM. In diabetic patients, the tear production, tear secretion and tear film stability are decreased [69]. Hyperglycaemia-induced AGE and sorbitol accumulation within the cells leads to cellular oedema and dysfunction, resulting in structural and functional damage of the lacrimal gland [69]. Additionally, diabetic neuropathy impairs the autonomic control of the lacrimal gland [70]. Consequently, both these factors decrease the capacity of the lacrimal gland to produce tears.

The basal and reflex tear secretion may also be diminished in diabetic patients [71]. The tear reflex has two reflex arcs that mediate the tear secretion: The motor arc stimulates blinking, and an autonomous arc produces secretion of tears. The impaired corneal sensation in diabetic patients compromises the tear reflex that eventually alters the production and secretion of tears [72].

Diabetic neuropathy also affects tear film stability. An inverse correlation was observed between tear film stability and the total peripheral neuropathy score [73]. The goblet cells of the conjunctiva together with the corneal and conjunctival epithelial cells secrete mucin, which contributes to the mucous layer of the tear film, providing a protective effect on the ocular surface. Mucin additionally forms glycocalyx, which promotes cell adhesion and makes the tear film hydrophilic, thus contributing to the tear film’s stability [69]. In diabetic patients, the number of goblet cells decreases, leading to the reduction of mucin production and the hydrophilicity of the tear film, resulting in tear film instability [74].

The DM-induced reduction in tear production, tear secretion and tear film stability may further degrade the quality of tear film in an affected eye and adversely impact the prognosis of NK. Schirmer’s test, tear break-up time and tear osmolarity allow for a detailed assessment of changes in the tear film [75], helping monitor disease progression and evaluate the efficacy of treatment.

#### 5.2.5. Corneal Confocal Microscopy

In recent years, IVCM has gained increasing importance to study the morphological changes of corneal nerves. IVCM provides good image contrast, with a lateral image resolution of 1–2 μm, the axial resolution of 5–10 μm, and magnification of 600–800 times [1,76]. The images obtained can be processed manually, in a semi-automated way or an automated manner using different imaging and analytic software to objectively evaluate corneal nerve metrics of the subbasal nerve plexus, including nerve fibre density, nerve branch density, nerve fibre length, total branch density, nerve fibre area, nerve fibre width, nerve fractal dimension, and nerve fibre tortuosity [40]. The quantitative nerve metrics help clinicians better understand the pathogenesis, disease severity, neuronal degeneration and regeneration patterns, as well as monitor disease progression in an objective manner in patients with diabetic corneal neuropathy. The relationship between IVCM findings on corneal nerves and DPN has been described in Section 4.2.

**Figure 3:**
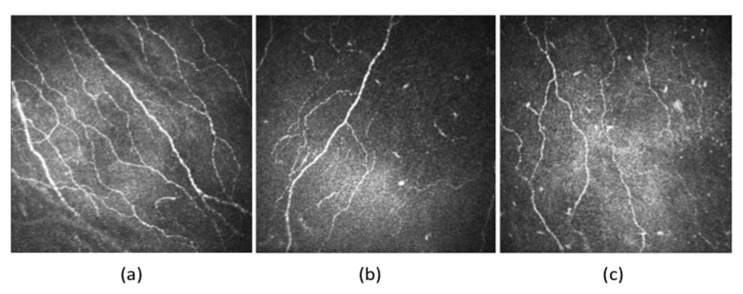
In-vivo confocal images of the subbasal nerve plexus of the (**a**) non-diabetic, (**b**) Type 1 diabetic and (**c**) Type 2 diabetic individuals. In both Type 1 and Type 2 diabetic patients, the corneal nerve fibre density, nerve fibre length, and total branch density are decreased compared to non-diabetic subjects. The nerves are more tortuous in patients with DM compared to controls.

**Figure 4:**
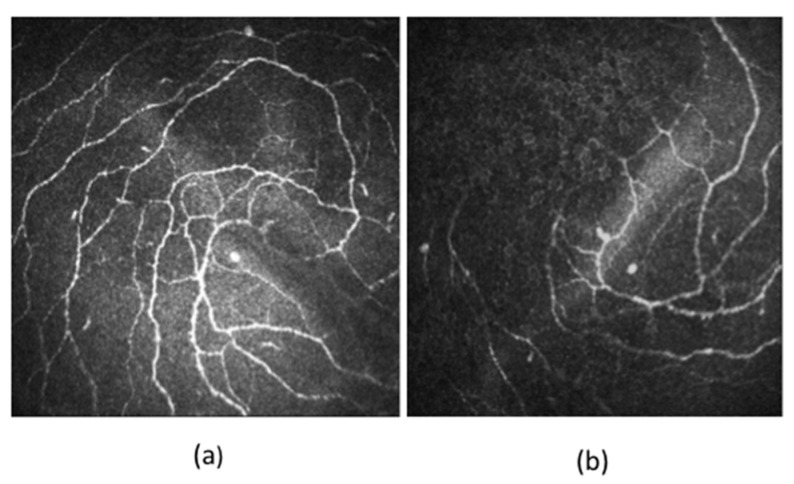
In-vivo confocal images of the inferior whorl of corneal nerves of the (**a**) non-diabetic and (**b**) diabetic individuals. The length and density of the inferior whorl fibres are decreased in patients with DM compared to non-diabetics.

A reduction of corneal nerve fibre length, nerve fibre density and nerve branch density, as well as an increase in nerve fibre tortuosity, was seen in patients with DM (Figure 3). Increased tortuosity represents ongoing corneal neuronal degeneration and regeneration during the clinical course of DM [10,77]. Some studies have also reported a significant reduction in nerve beading frequency, indicating reduced metabolic activity and increased risk of neuronal damage in patients with DM [10,78]. The nerve fibre length and nerve fibre density of the subbasal inferior whorl of the corneal nerves may also be decreased in DM (Figure 4), presenting as an early manifestation in comparison to the nerve changes in the central cornea. It could be considered as the optimal imaging site in diabetic patients to improve the diagnostic performance of IVCM scans [63,79].

Several studies have been conducted to determine the correlation between the IVCM findings of corneal nerves and HbA1c level. The results have been contrasting [10]. While some authors reported that corneal nerves parameters correlated inversely with HbA1c level, others concluded that there was no correlation [10]. Hence, the impact of the HbA1c level on corneal nerves warrants more investigation with controlled study design. Furthermore, longer duration of DM is a risk factor for the progression of corneal neuropathy, with an inverse correlation between the duration of DM and the deterioration of key corneal nerve parameters, e.g., corneal nerve fibre length, density, and branch density [10].

## 6. Management of Diabetic Keratopathy

Good glycaemic control is able to improve corneal neuropathy [80]. This observation was supported by the results of the studies that were conducted by Azmi et al. [81,82] and Jia et al. [83]. They demonstrated that corneal nerve parameters improved following glycaemia amelioration in patients with type 1 [81] and type 2 DM [82,83]. Likewise, the improvement of glycaemic control (HbA1c) was found to correlate significantly with the increase in corneal nerve fibre density [84]. These findings suggest that systemic control of DM is the cornerstone in the management of diabetic corneal neuropathy. While the goal of systemic therapy is to achieve optimal glycemic control, local treatment aims at maintaining a healthy tear film together with an ocular surface with intact epithelium. The treatment options depend on the severity of keratopathy, and a step-wise approach is generally suggested, as described below.

### 6.1. Stage 1 NK

The therapeutic goals of stage 1 NK management include: (1) To promote corneal epithelial integrity and avoid its breakdown; (2) to improve health and quality of corneal epithelium; and (3) to prevent progression to stage 2 NK.

For stage 1 NK, discontinue all topical medications if possible, except preservative-free artificial tears or ointment to avoid toxic keratopathy from preservatives. Reducing concurrent inflammation with the use of topical non-preserved steroids, non-steroidal anti-inflammatory drugs (NSAID) or ciclosporin is also indicated. However, topical steroids should be used with caution because they may increase the risk of secondary microbial infections [85]. Likewise, instillation of topical NSAID may lead to the increased propensity of corneal melting and perforation as they inhibit stromal healing, hence should be cautiously administered [63,86]. It is also advisable to treat ocular surface diseases, e.g., exposure keratitis, dry eye and limbal stem cell deficiency, as well as lid abnormalities, as these pathologies have negative impacts on the ocular surface, thus worsening the prognosis of NK. Punctal occlusion helps to increase the retention of natural tears and promote the corneal healing process [87]. While punctal occlusion may be achieved with various methods, such as temporary or short-acting collagen plugs, permanent punctal or canalicular plugs, as well as laser cautery or surgical ligation, they may also be associated with complications, such as punctal extrusion, local irritation, and pyogenic granuloma formation [88,89,90]. In addition, the timing of the insertion of punctal plugs is critical for optimal results. The insertion should be avoided during active inflammation as it may lead to accumulation of tears that are concentrated with pro-inflammatory cytokines, thus impeding corneal wound healing [91].

### 6.2. Stage 2 NK

The treatment goals of stage 2 NK include re-epithelialisation of the denuded stroma and prevention of corneal infections and stromal lysis. These intervention aims can be achieved by the following therapeutic modalities.

#### 6.2.1. Debridement

In some patients, the leading edge of the healing epithelium may become heaped, which may prevent corneal epithelial migration across the epithelial defect. Debridement of rolled edges of the neurotrophic ulcer promotes corneal re-epithelialisation by triggering the healing response in the surrounding epithelium [92].

#### 6.2.2. Therapeutic Contact Lenses

Corneal or scleral therapeutic contact lenses can promote ocular surface integrity as they (1) decrease the trauma from the eyelids (2) create a reservoir of therapeutic agents and lubricants between the contact lens and cornea, as well as increase their retention time on the ocular surface. However, therapeutic contact lenses should be cautiously used in patients with NK as reduced, or absent corneal pain sensation might mask an early sign of corneal infections [54].

#### 6.2.3. Autologous Serum Eye Drops

Over the last decade, there has been an increased interest in the use of autologous serum eye drops to treat persistent corneal epithelial defects in patients with stage 2 NK who are unresponsive to conventional medical treatment because of superior efficacy in comparison with traditional lubricants [54]. Autologous serum eye drops contain important components of the tear film, such as vitamin E, vitamin A, platelet-derived growth factor, NGF, fibroblast growth factor, fibronectin, SP, epidermal growth factor, and insulin-like growth factor (IGF), [93] enhancing the proliferation and migration of the corneal epithelial cells. Their efficacy and safety for treating persistent corneal epithelial defects have been established, with a healing rate of 46.7–68.0% within one month of initiating the therapy [94,95,96].

#### 6.2.4. Platelet-Rich Plasma

Platelet-rich plasma (PRP) also contains biologically active proteins and growth factors and has been used as a therapeutic agent to promote wound healing and regeneration of the ocular surface. Some studies have demonstrated that PRP had better efficacy than autologous serum eye drops as it healed those persistent epithelial defects where topical autologous serum drops had failed [54,97].

#### 6.2.5. Amniotic Membrane Transplantation (AMT)

**Figure 5:**
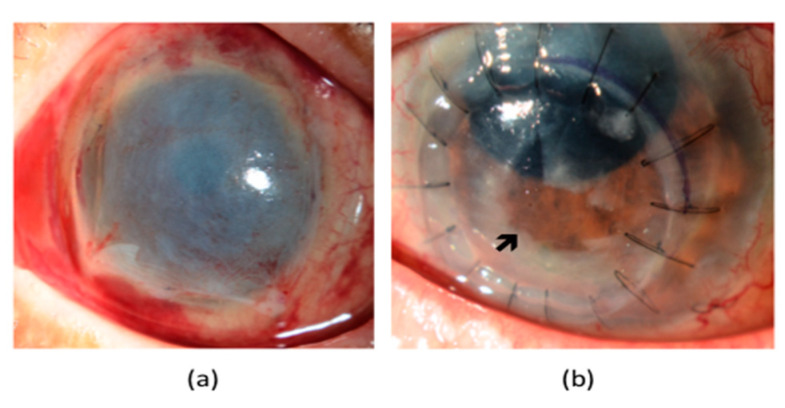
Surgical management of NK. (**a**) Amniotic Membrane Transplantation (**b**) Lamellar Corneal Transplant. The lack of trophic support, due to surgery-induced corneal denervation may impair epithelial wound healing and result in a persistent corneal epithelial defect, as shown by the black arrow.

Amniotic membrane (AM) is a biological tissue that has been used by ophthalmologists for the management of a variety of ocular conditions, including neurotrophic corneal ulcers (Figure 5a). While AM is routinely applied by using fibrin glue or sutures, innovative devices, such as OmniLenz^®^ and ProkeraTM, which consist of a combination of AM and a contact lens that can easily be slipped into the eye, have also gained attention [54].

AM has three distinct layers: Epithelium, basement membrane, and stroma. The basement membrane comprises of collagen IV and VII, laminin and fibronectin that facilitate epithelial cell adhesion. In contrast, the stromal part contains various growth factors, anti-inflammatory factors, tissue-inhibitors of matrix metalloproteinases and anti-angiogenic factors. The complex interaction of biological mediators assists in the resolution of neurotrophic corneal ulcers [54,98]. Several studies have established the efficacy of AMT in the treatment of neurotrophic corneal ulcers that were unresponsive to conventional therapy [54,99]. Kruse and colleagues showed that neurotrophic ulcers healed within four weeks after AMT. However, the disintegration of the AM occurred faster than the healing of the corneal ulcer. Therefore multiple layers of AM were suggested for complete re-epithelialisation of the denuded stroma [100]. Similarly, Chen et al. demonstrated that AMT achieved rapid epithelialisation in 76% of the eyes within 16 days. However, some of the patients needed adjunctive therapy, such as tarsorrhaphy for complete epithelial healing [101]. These studies showed that single layer AMT in isolation might not be effective enough for severe neurotrophic corneal ulcers, and multilayer AMT or combining AMT with other therapies, i.e., preservative-free lubricants, bandage contact lenses, tarsorrhaphy, should be considered for rapid and complete corneal re-epithelialisation in severe cases.

#### 6.2.6. Eyelid Closure

Eyelid closure to narrow the interpalpebral fissure is the cornerstone in the management of NK, to eliminate blink-related trauma to the healing epithelium and to reduce evaporative tear loss [102]. Moreover, it provides a tear reservoir that keeps the cornea moist and protects it from the environmental pathogens. Although eyelid closure could be achieved by non-surgical techniques, such as adhesive tapes, pressure patching and botulinum toxin injection-induced ptosis, these techniques have some limitations, such as the need for repeat botulinum toxin injection for a prolonged effect [103]. Hence, the surgical approximation of the upper and lower lids by tarsorrhaphy provides a more definitive closure of the eyelids. Tarsorraphy can be partial or complete, as well as temporary or permanent depending on the severity of the NK [104]. Even if permanent, tarsorrhaphy can be opened conveniently when the healing of the ocular surface is achieved. It should be considered in the cases of persistent corneal epithelial defects that are refractory to medical treatment and/or non-surgical interventions of eyelid closure [105].

#### 6.2.7. Conjunctival Flaps

Conjunctival flaps may be useful in the management of non-healing corneal epithelial defects where medical management has failed because they provide vascular blood supply and serum-derived growth factors to the non-healed area [106,107,108]. This facilitates the inhibition of the ongoing inflammatory process and promotes epithelial healing. However, disadvantages of conjunctival flaps include reversibility issues, flap retraction and risk of corneal perforation under the flap [106,107,108]. Despite disadvantages, conjunctival flaps, such as Gundersen’s flap and pedicle flap, are commonly used to treat non-healing neurotrophic ulcers [109,110].

#### 6.2.8. Nerve Growth Factor (NGF) Eye Drops

NGF eye drops have become a promising therapeutic modality for the treatment of NK. Clinically, it promotes corneal epithelial healing, improves tear function and stimulates corneal nerves regeneration [111]. Several studies have established the safety and efficacy of NGF eye drops in the management of NK [112,113,114]. A multi-centre clinical trial recently demonstrated the efficacy of topical rhNGF in patients with moderate or severe NK that were unresponsive to conventional medical treatment. The study participants either received topical rhNGF 20 μg/mL (Cenegermin) or vehicle six times daily in the affected eye for eight weeks. Besides a good safety profile, the results showed that rhNGF eye drops had higher rates of corneal healing than the vehicle (69.6% and 29.2%, respectively). rhNGF-treated patients also presented a significant reduction in the size of corneal epithelial defect and disease progression rates compared to vehicle-treated patients [115]. In another study, topical rhNGF eye drops improved the corneal subbasal nerve density in patients with NK, suggesting the beneficial effect of rhNGF eye drops on corneal nerves regeneration [116].

#### 6.2.9. Prevention of Infection and Stromal Lysis

In neurotrophic corneas, a secondary microbial infection could delay the healing of persistent corneal epithelial defects (Figure 2c). It may be worthwhile to start prophylactic preservative-free broad-spectrum topical antibiotics to prevent secondary microbial infections. Wherever possible, topical aminoglycosides, e.g., gentamicin, should be avoided because they cause drug toxicity [117] and may aggravate NK. In the event of corneal infection, stromal degradation and melting can occur, due to the release of proteolytic enzymes from the recruited inflammatory cells, as well as the activation of pro-inflammatory and tissue-destructive cascades. Topical and/or systemic administration of tetracyclines, macrolides, acetylcysteine or ascorbate has been used to prevent corneal melting because they inhibit matrix metalloproteinases (MMPs) and collagen degradation, downregulate neutrophil collagenase expression, suppress neutrophil degranulation and scavenge ROS [118,119,120].

### 6.3. Stage 3 NK

NK could progress to stage 3, where patients may end up with a corneal perforation and/or corneal stromal melting. Hence, the management aims at preventing corneal stromal lysis and corneal perforation [63]. Although cyanoacrylate glue application followed by the use of soft bandage contact lens or AMT has been shown to be effective in sealing a perforation if it is <3 mm, larger defects usually require a conjunctival flap or lamellar/penetrating keratoplasty [63]. While tectonic keratoplasties restore the structural integrity of the cornea and provide better vision than AMT or conjunctival flaps if the visual axis is involved (Figure 5b), they carry risks of graft rejection and failure. The lack of trophic support, due to corneal denervation resulting from surgery may also retard epithelial wound healing, which needs to be carefully attended to. Boston keratoprosthesis implantation can also be considered for visual rehabilitation of the patients that have undergone multiple failed surgical interventions for severe NK [92].

In addition, topically administered biological polymers, such as matrix-regenerating agent (RGTA), have also been proposed for the management of severe NK. RGTA promotes corneal healing by providing binding sites for growth factors and avoids stromal proteolysis [121].

## 7. Future Perspectives

Clinicians and researchers are striving to identify potential biomarkers and new treatment strategies for diabetic corneal neuropathy. The advancements in corneal imaging and development of innovative clinical tests may help the eye care professionals to detect corneal neuropathy early and to monitor its progression.

### 7.1. Tear Biomarkers for Corneal Neuropathy

Tears are easily obtainable, and the use of tear biomarkers to predict or stratify an ocular condition is gaining significant importance. A correlation has been found between the tear biomarkers that are involved in corneal health, such as IGF-1 and SP and IVCM findings in diabetic patients [72,122,123].

#### 7.1.1. Insulin-Like Growth Factor 1 (IGF-1) and Insulin-Like Growth Factor Binding Protein-3 (IGFB-3)

IGF-1 is an important component of the tear film proteome and has potent neuroprotective functions [124]. In diabetics, the protective effect of IGF-1 on corneal nerves is impaired, as IGF-1 is sequestered by IGFB-3, whose expression is upregulated, due to hyperglycaemia and its associated oxidative stress [125]. This shift in the IGF-1/IGFBP-3 balance may result in apoptosis and degradation of corneal nerves [125]. The IGFB-3 level in tears was found to be 3.5-fold higher in diabetics compared to non-diabetics and was highly correlated with deterioration of corneal nerves [123]. Therefore, IGFB-3 may serve as a biomarker for the severity of diabetic corneal neuropathy.

#### 7.1.2. Substance P (SP)

Tear SP may be another potential biomarker for diabetic corneal neuropathy. SP is a key neuropeptide that is secreted by corneal nerves. It maintains corneal epithelial integrity by mediating epithelial migration, proliferation and differentiation via the neurokinin-1 receptor (Table 1). In diabetics, SP plays a role in the recovery of corneal sensitivity by modulating neurogenic inflammation [36,56,122]. The SP concentration in tears was significantly reduced in type 1 diabetic patients with DPN in comparison to those without DPN and healthy individuals and correlated with both corneal nerve fibre density, as well as peripheral neuropathy [122].

#### 7.1.3. Inflammatory Tear Biomarkers

Recently, Meera and colleagues explored the role of 31 tear biomarkers for screening of DPN. MMP-9 and tumour growth factor-α (TGF-α) levels in tears were higher in patients with DPN compared to patients without DPN, but the difference was not significant. The authors concluded that inflammatory tear biomarkers were regarded as suboptimal standalone tools for detecting DPN as the AUC was only 65%. However, combining tear analysis (MMP-9 and TNF-α) with corneal esthesiometry measurement (cut off 5.8 mm) could improve the AUC to 84% [126].

### 7.2. Emerging Therapies for Diabetic Corneal Neuropathy

With growing interest in the treatment of diabetic corneal neuropathy, development of new therapies to promote and maintain ocular surface integrity, as well as corneal neuronal health, is imperative. The systemic and topical therapeutic approaches, as well as gene and cell-based therapies that have been tested and suggested in recent years, are summarised in Table 3. However, researchers have proposed these treatment options by extrapolating results from experiments that were mostly conducted in animal models; hence, further investigation and clinical studies are required to attest their safety and efficacy in patients with DM.

### 7.3. Innovative Clinical Tools

Corneal confocal imaging is a rapid and non-invasive tool to evaluate the severity of diabetic corneal neuropathy, as well as response to treatment. However, costly equipment, limitations of the current image acquisition systems and analytic technology, have limited the widespread adoption of IVCM in routine clinical practise, as well as screening programs for diabetic neuropathy. The commercially available IVCM scans a small area of interest (<500 μm^2^), hence scanning on multiple areas may be required if clinicians intend to depict the nerve network over the whole cornea. Wide-field or large-area corneal scanning, as well as montaging techniques, have the potential to circumvent this [140]. Moreover, efforts would be required to improve the current approach of image analysis, to be less time-consuming, as well as to better detection and quantification of finer, out-of-focus and low-contrast nerve fibres, especially on images with a relatively ‘noisy background’. An artificial intelligence-based deep learning algorithm with data augmentation has been developed to improve the quantification of corneal subbasal nerve plexus for the diagnosis of diabetic neuropathy [141]. The availability of post-scan image-processing and image-enhancing tools may also enhance its performance.

Furthermore, the scale of conventional clinical assessment, such as tear break-up time and corneal sensitivity with Cochet-Bonnet esthesiometer, maybe too crude and not sensitive enough to detect early corneal neuropathy or to monitor the subtle progression of DK. Improvement in the assessment tool for corneal sensitivity, or other advanced clinical tests for the evaluation of ocular surface, such as tear film stability analysis system, TearScope (Keeler, Windsor, UK), Oculus Keratograph 5M (Oculus, Arlington, WA, USA) and LipiView II (TearScience, Morrisville, NC, USA) that reliably determine the production and stability of the tear film, maybe useful.

## 8. Conclusions

Diabetic keratopathy affects 47–64% of patients during the clinical course of DM and can have a significant economic burden. Chronic hyperglycaemia and its associated metabolic changes result in corneal neuronal degeneration. Recent advancements in corneal nerve imaging and software have allowed quantitative evaluation of the morphology of corneal nerves, and emerging scientific evidence has established that DM-induced corneal neuropathy correlates with changes in peripheral nerves. Hence, the corneal nerve plexus may be regarded as a surrogate marker of DPN. The clinical manifestations of diabetic corneal neuropathy vary with disease severity and range from decreased corneal sensitivity to neurotrophic ulcers that have a risk of vision-threatening corneal infections. The management of DK depends on its severity, and a step-wise approach is generally suggested. Future work would focus on exploring the potential biomarkers and treatment strategy, as well as new innovative clinical tests and imaging modality to detect diabetic corneal neuropathy early and to monitor its sub-clinical progression.

## Figures and Tables

**Table 1:** Summary of the neurotrophic factors produced by the corneal nerves.

Categories	Neuromediator	Mechanism of Action	Functions [1,37,54]
Neurotrophins	NGF	The binding of NGF with TrkA decreases NF-κB nuclear translocation, inhibits GSK3 activity, as well as enhances the activation of the PI3K/Akt pathway to elicit downstream events [51].	(1) Maintains neural homeostasis and promotes corneal neuronal regeneration. (2) Reduction of neuroinflammation. (3) Maintains corneal nerve density and modulates corneal sensitivity. (4) Corneal nociception. (5) Enhances corneal wound healing by stimulating epithelial cell migration, colony formation and proliferation.
	NT-3	NT-3 interacts with TrkC and activates GTPase, Ras, phosphatidylinositol 3-kinase and PLC-g1 to regulate neuronal survival and differentiation via activation of mitogen-activated protein kinases [55].	(1) Essential factor of sensory and sympathetic corneal neurons. (2) Regulates corneal neuronal regeneration.
Neuropeptides	SP	SP acts on Neurokinin-1 receptor and activates EGFR together with Sirt1 and Akt-signalling pathways to promote corneal epithelial wound healing [56].	(1) Modulator of corneal epithelial proliferation, migration and adhesion. (2) Inhibits apoptosis of corneal epithelial cells. (3) Recovery of corneal sensation. (4) Improvement of mitochondrial function in corneal epithelial cells.
	CGRP	CGRP is released from corneal sensory neurons in response to pain stimulus, and it upregulates the expression IL-8, which is a neutrophil chemotactic protein [57].	(1) Maintenance of corneal epithelial integrity by facilitating epithelial cell migration and differentiation. (2) Corneal nociception. (3) Vasoactive effects. (4) Modulator of innate immunity.
	NPY	NPY mediates its response by binding to NPY 2 receptor that inhibits adenylate cyclase activity and increases intracellular Ca^2+^ levels through pertussis toxin-sensitive G proteins [58].	(1) Stimulator of angiogenesis and angiogenesis-dependent wound healing. (2) Anti-inflammatory effects on the cornea.
	VIP	VIP performs its functions by downregulating pro-inflammatory and upregulating anti-inflammatory Toll-like receptors in a cAMP-dependent fashion [59].	(1) Promotes corneal neuronal regeneration. (2) Anti-inflammatory effects on the cornea. (3) Promotes neurotrophin production.
Neurotransmitters	Catecholamines (epinephrine and norepinephrine)	Catecholamines bind to adrenergic receptors present on the plasma membrane of effector cells. This would either promote or inhibit adenylyl cyclase-mediated intracellular cAMP production, or influence PKC signalling mechanism, to modulate downstream events [60].	(1) Corneal epithelial cell migration and proliferation. (2) Transcellular transport.
	Ach	Ach activates both muscarinic and nicotinic receptors, allowing gated ion channels to open for the influx of Na+ and Ca^2^+ and efflux of K+. It also regulates the activities of phosphatases and protein kinases [61].	(1) Maintains an ionic gradient during nerve impulse conduction. (2) Corneal epithelial cell DNA synthesis and epithelial cell migration. (3) Corneal stromal keratocytes proliferation. (4) Inhibits corneal fibrosis and apoptosis.

NGF, Nerve growth factor; TrkA, Tropomyosin receptor kinase A; NF-κB, Nuclear factor-κB; GSK3, Glycogen synthase kinase 3; PI3K/Akt, Phosphatidylinositol-3-Kinase/Protein Kinase B; NT-3, Neurotrophin-3; TrkC, Tropomyosin receptor kinase C; GTPase, Guanosine triphosphatase; Ras, Rat sarcoma; PLC-g1, Phospholipase C; gamma 1; SP, Substance P; EGFR, Epidermal growth factor; Sirt1, Sirtuin 1; CGRP, Calcitonin gene-elated peptide IL-8, Interleukin-8; NPY, Neuropeptide Y; VIP, Vasoactive intestinal peptide; cAMP, Cyclic adenosine monophosphate; Ach, Acetylcholine.

**Table 2:** Common causes of neurotrophic keratopathy [54].

1. *Genetic Causes* [64]
Familial corneal hypoaesthesia
Mobius syndrome
Goldenhar-Gorlin syndrome
Familial dysautonomia (Riley-Day Syndrome)
2. *Ocular Causes*
Chemical burns
Post-herpetic infections
Acanthamoeba infections
Toxicity of topical anaesthetics and topical drugs (e.g., diclofenac sodium, etc.)
Chronic ocular surface inflammation
Contact lens wear
Corneal dystrophies (Lattice or granular dystrophies)
Orbital Neoplasia
3. *Systemic Causes*
DM (diabetes mellitus)
Multiple sclerosis
Alzheimer’s disease
Parkinsonism
Leprosy
Vitamin A Deficiency
Amyloidosis
Neoplasms and aneurysms of the central nervous system
Cerebral vascular accidents
4. *Surgical Causes* [54]
Refractive surgery [65]
Corneal cross-linking
Panretinal photocoagulation for diabetic retinopathy
Post-cataract surgery
Post-vitrectomy
Post-neurosurgical procedures (e.g., surgery for trigeminal neuralgia, acoustic neuroma etc.)
Iatrogenic injury to the trigeminal nerve

**Table 3:** Emerging therapeutic approaches to diabetic corneal neuropathy.

Therapeutic Agent	Mechanism of Action	Route of Administration	Experimental Evidence
IGF-1	Growth factor	Topical	IGF-1 treatment protected against the corneal nerve damage and improved corneal subbasal nerve density in type 2 diabetic mice [127].
Sema3a	Membrane-bound axon-guidance protein	Intrastromal Sema3a pellet implantation	Sema3a induced neuronal regeneration in the injured mice corneas [128].
Sema7a	Membrane-bound axonal growth promoter and immune regulator	Sema7a pellet implantation under the corneal flap after lamellar nerve-transection surgery	Sema7a supplementation promoted nerve regeneration and influenced inflammatory processes in the mice cornea [129].
Ilepatril	Vasopeptidase inhibitor	Systemic	Ilepatril prevented the loss of motor and sensory nerve conduction velocity, as well as the decrease in intraepidermal nerve density, in type 1 diabetic rats [130].
Enalapril	ACE inhibitor	Systemic	Treating diabetic rats with the combination of enalapril, α-lipoic acid and menhaden oil reversed diabetic corneal and peripheral neuropathy [131].
Resolvin-D1	Anti-inflammatory eicosanoid	Systemic	Resolvin-D1, together with menhaden (fish) oil supplementation, reduced the degeneration of corneal and peripheral nerves in diabetic rats [132].
Naltrexone	Long-acting opioid antagonist	Topical/Systemic	Naltrexone promoted corneal epithelial wound repair and restored corneal sensitivity in type 1 and 2 diabetic rats [133,134].
Fenofibrate	PPARα agonist	Systemic	Oral administration of fenofibrate protected the corneal nerves from degeneration in streptozotocin-induced diabetic rats and mice [135].
miR-146Aa	Regulates gene expression by repressing translation	Topical	Targeted inhibition of miR-146Aa promoted wound healing by enhancing the migration of limbal epithelial cells in human diabetic corneas; however, this has been observed in organ cultures only [136].
Experimental Gene Therapy	c-met proto-oncogene overexpression and/or cathepsin F and MMP-10 gene silencing	Vector delivery	Adenoviral gene therapy enhanced corneal epithelial wound healing and normalised stem cell marker expression in organ-cultured human diabetic corneas [137,138].
MSCs	TSG-6 mediated corneal epithelial wound healing	Subconjunctival injections of bone marrow-derived MSCs	Locally transplanted MSCs promoted the healing of corneal epithelial wounds in diabetic mice via activation of corneal epithelial stem/progenitor cells [139].

IGF-1, Insulin-like growth factor 1; Sema3a, Semaphorin3a; Sema7a, Semaphorin7a; ACE, Angiotensin-converting enzyme; PPARα, Peroxisome proliferator–activated receptor α; miR, microRNA; c-Met, Tyrosine-protein kinase Met; MMP-10, Matrix metalloproteinase-10; MSCs, Mesenchymal stem cells; TSG-6, Tumor necrosis factor-α-stimulated gene/protein-6.
